# American Football Headgear Impairs Visuomotor Drill Performance in Division I NCAA Football Athletes

**DOI:** 10.3390/jfmk9030169

**Published:** 2024-09-18

**Authors:** Christopher G. Ballmann, Rebecca R. Rogers

**Affiliations:** 1 Department of Human Studies, University of Alabama at Birmingham, Birmingham, AL 35294, USA; 2 Department of Physical Therapy, University of Alabama at Birmingham, Birmingham, AL 35294, USA; 3 Center for Exercise Medicine, University of Alabama at Birmingham, Birmingham, AL 35294, USA; 4 Center for Engagement in Disability Health and Rehabilitation Sciences, University of Alabama at Birmingham, Birmingham, AL 35294, USA; 5 Department of Family and Community Medicine, University of Alabama at Birmingham, Birmingham, AL 35294, USA; rrrogers@uab.edu

**Keywords:** helmet, facemask, visor, eyeshield, vision

## Abstract

**Background/Objectives:** Previous evidence has shown that American football headgear (e.g., facemasks, visors/eye shields) differentially impairs reaction time (RT) to visual stimuli, most notably in peripheral fields of view. However, this has only been established with stationary RT testing, which may not translate to gameplay situations that require gross motor skills. Therefore, the purpose of this study was to build upon previous findings to elucidate the effects of various American football headgear on gross motor visuomotor drill performance. **Methods:** Division 1 NCAA football players (n = 16) with normal/corrected-to-normal vision participated and completed two experiments (EXP), each with differing conditions: EXP1- Varying facemask reinforcement and EXP2- Varying visor/eye shield light transmittance. In EXP1, participants completed an agility test for the following conditions: baseline/no helmet (BL), helmet + light (HL), helmet + medium (HM), and helmet + heavy (HH) face mask reinforcement. In EXP2, participants completed an agility test for the following conditions: baseline/no helmet (BL), helmet + clear visor (HCV), helmet + smoke-tinted visor (HSV), and helmet + mirrored visor (HMV). For each condition in EXP1 and EXP2, participants completed a reactive agility task using a FITLIGHT trainer system where five poles were equipped with a total of ten LED sensors and were placed in a semi-circle 1 m around a center point. Participants were asked to step and reach with their hands to hit each ten lights individually as fast as possible upon illumination. Each reactive agility test was repeated for a total of three attempts. **Results:** Average reaction time was analyzed and compared between conditions and according to visual fields of interest (e.g., central vs. peripheral). Results from EXP1 showed that compared to BL, reactive agility was worsened by HL (*p* = 0.030), HM (*p* = 0.034), and HH (*p* = 0.003) conditions. No differences between facemask conditions existed for overall performance (*p* > 0.05). For EXP2, HCV (*p* < 0.001), HSV (*p* < 0.001), and HMV (*p* < 0.001) conditions resulted in worsened reactive agility performance compared to BL. No differences between visor conditions existed for overall performance (*p* > 0.05). **Conclusions:** Overall, these findings suggest that American football headgear impairs reactive agility, which could result in worsened game performance and safety. Future studies investigating training strategies to overcome impairments are warranted.

## 1. Introduction

The development of protective headgear in American football (AF) has seen significant changes since the sport’s inception in the 1800s [1]. Protective headgear debuted in collegiate football in 1896 and was constructed from various types of animal hide and leather. These primitive designs were effective at protecting the player’s ears from blunt auricular trauma but offered little protection from blunt force trauma to the head. As the sport gained popularity and saw increases in participation, reports of accidents, injuries, and fatalities from head and neck injuries became more prevalent. By the middle of the 20th century, leather helmets were replaced by heavy gauged plastic with internal padding and were equipped with reinforced facemasks. Modern-day football helmets are notably overbuilt compared to predecessors with polycarbonate shells and carbon steel facemasks in an effort to lower head and neck injuries, a design change in which the efficacy remains debated [2]. Orbital injuries have also remained prevalent during the development of AF headgear, leading to the implementation of additional eye protection, often termed “visors”. Visors are also commonly made from polycarbonate but allow for varying degrees of light transmittance, effectively protecting the eyes from openings in the facemask while still giving athletes visibility. While some organizations recommend visors for all players [3], they are suggested to be especially important for players with pre-existing conditions that increase their risk for ocular complications. Despite the increased protection, the bulkier designs of AF headgear obstruct the visual field and reactive ability. Since clear vision and reactive ability are fundamental for optimal performance and safety during gameplay, understanding how modern protective football headgear affects a player’s ability to respond to visual stimuli is essential for player safety and the development of equipment regulations [4].

Decades of empirical evidence have shown that AF protective headgear impairs visual fields [5,6,7,8,9]. In the 1960s, Schneider et al. were among the first to show detailed mapping of visual impairments from varying types of facemasks [5]. While AF headgear technology has progressed, similar findings of impaired vision and visuomotor performance have been reported in modern equipment. Kramer et al. reported that eye–hand coordination task performance was hindered in recreationally active individuals whilst wearing a modern AF helmet versus no helmet [10]. Miller et al. showed that wearing a helmet with a standardized facemask significantly impairs reaction time and target detection in collegiate football players [9]. A follow-up study confirmed these impairments further and showed that helmets equipped with heavily reinforced facemasks exacerbate visuomotor performance decrements [6,7]. While the use of visors with or without various tinting and light transmittance has been validated for safety consideration [11], the impact of how they influence visuomotor ability has yet to be fully determined. RT in response to peripheral visual stimuli while wearing an AF helmet appears to remain mostly unaltered by the addition of a clear or non-tinted visor [9]. However, helmets equipped with dark or mirror-tinted visors have been shown to impair the reactive ability of healthy football players, most notably in peripheral fields [6]. Taken together, even AF headgear with modern advances has been shown to negatively impact the visual field and reactive motor ability. 

Maintaining clear vision and view is important for in-game performance and safety. While previous investigations have clearly noted impairments in reactive ability in AF players while equipped with headgear [5,6,7,9], almost all testing was completed with the athlete remaining stationary. AF is a highly dynamic sport that requires a unique mixture of strength, decision-making, visual processing, and reactive ability. Thus, previous findings with stationary protocols may not readily translate to gameplay where dynamic visuomotor performance is of the utmost importance. While little evidence exists in this regard, Shelly et al. attempted to attenuate headgear-induced decrements in dynamic visuomotor performance by creating a new AF helmet prototype with a greater visual field compared to more standard models [8]. The prototype design improved players’ subjective perceptions of vision impairment but still compromised objective measurements of dynamic visuomotor performance in collegiate athletes [8]. 

The National Collegiate Athletic Association (NCAA) currently gives 15 different examples of legal facemasks to be used in gameplay in their rules and interpretations guidelines [12]. Furthermore, tinted visors and the use of eye shields, while typically legal only under medical guidance, continue to be used in gameplay. While previous investigations have suggested different types of facemasks and visors may impair visuomotor ability [6,7], it remains unknown if impairments translate to dynamic movements and reactive visuomotor ability, which are commonly employed in competitive gameplay. Given previous reports have suggested that enhancements in visuomotor ability may lower concussion incidence in collegiate AF players [13], understanding how facemasks and visors influence dynamic visuomotor ability has important implications for player performance and safety. The purpose of this study was two-fold: (1) to determine how varying levels of AF facemask reinforcement influence dynamic visuomotor performance; and (2) to determine if the addition of a clear, tinted, or mirrored visor to an AF helmet influences dynamic visuomotor performance.

## 2. Materials and Methods

### 2.1. Study Design

The current study utilized a randomized and counterbalanced repeated-measures design to determine the effects of AF facemask and visor type on dynamic visuomotor ability. Two experiments (EXP1 and EXP2) were completed in NCAA Division I AF players with different interventions: EXP1) Varying facemask reinforcement, EXP2) Varying visor tint and visual light transmittance (VLT). For each condition in EXP1 and EXP2, players completed three attempts of a dynamic visuomotor test consisting of reacting and moving to deactivate LED timing sensors in various fields of view. Response times were compared between conditions and were recorded from the illumination to the deactivation of each sensor. Visits were separated by a minimum of 24 h and completed approximately at the same time of day. Baseline conditions were included in both experiments to counteract possible day-to-day differences in response times. Written and informed consent were obtained by each participant prior to testing. All experimental procedures and protocols were approved by the local institutional ethical committee (EXPD-HP-21-S-26) and complied with guidelines under the Declaration of Helsinki.

### 2.2. Participants

The appropriate sample size was determined using an a priori power analysis and comparison to previous literature [6,8,9]. A previous investigation on AF facemasks/visors and reaction time from our lab showed response time decrements with a partial η^2^ = 0.115 or an f effect size = 0.36 [6]. For the power analysis, the following parameters were utilized to compute sample size using G*power V 3.1.9.4 software: repeated-measures ANOVA, f = 0.36, α = 0.05, and β = 0.80. This resulted in a minimum sample size of n = 16 with a predicted observed power of β = 0.85. Accordingly, Division I NCAA male athletes (n = 16; age = 20.4 years ± 1.3; height = 181.3 cm ± 6.2; body mass = 91.2 kg ± 15.1; playing experience = 11.1 years ± 4.0) were recruited and volunteered to participate. Specialty breakdown of players included offense (n = 8), defense (n = 6), and special teams (n = 2). To confirm normal or corrected-to-normal visual acuity, all participants underwent a vision screening using a Snellen eye chart from a distance of 6 m [6]. To be included in the study, participants needed to meet the following criteria: active participation on a Division I NCAA football roster in the previous year, no concussions or eye injuries within the past 6 months, and no medical history of non-correctable vision abnormalities or conditions. Before testing, participants were instructed to avoid caffeine, nicotine, and alcohol for at least 12 h and to abstain from vigorous activity for 24 h [11]. Participants were unaware of any hypotheses related to the study. 

### 2.3. Dynamic Visuomotor Test

**Figure 1:**
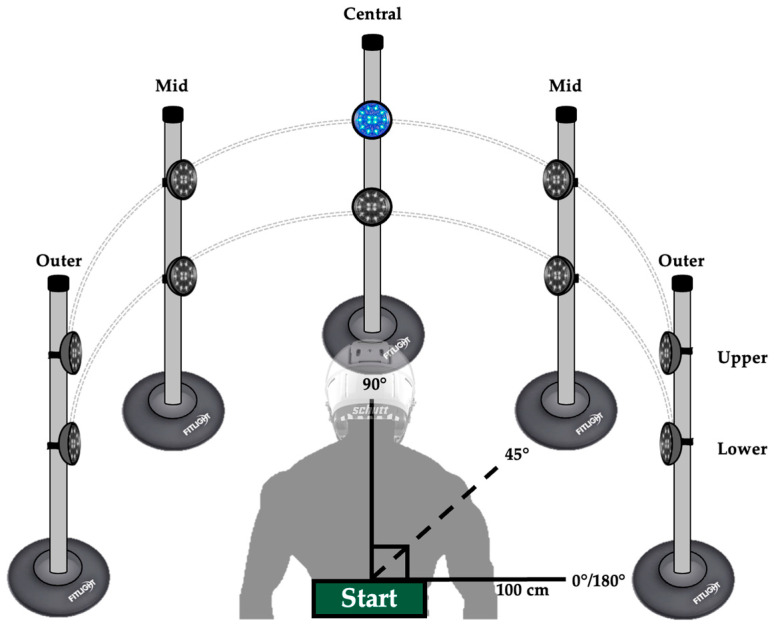
Diagram of the dynamic visuomotor test. Participants stood at a centralized starting point and were asked to step and deactivate LED timing sensors situated on poles in a semi-circle as quickly as possible. Upper sensors were 85 cm from the floor while lower were 42 cm. Each of the 10 lights illuminated in a random order for a total of 2 times (20 total attempts). Participants were given 1 s to return to the starting point after each sensor deactivation. The test was completed a total of 3 times with 3 min of rest in between.

The dynamic visuomotor test was adopted from Shelly et al. and is shown in Figure 1 [8]. A FITLIGHT timing system (FITLIGHT Sports Corp, Aurora, ON, Canada) was used to detect response time. The FITLIGHT system uses Bluetooth LED light sensors within a puck-shaped enclosure equipped with low-level infrared beams. Response time was measured from the moment the LED lights illuminated to when the infrared beams were broken when the participant made contact, which “deactivated” the LED sensor. The use of FITLIGHTs in this manner has been rigorously tested and confirmed for validity and reliability [14]. For the dynamic visuomotor test, FITLIGHT sensors were attached to five vertical poles in a semi-circle from a set start point. Poles were 1 m from the starting point around the participant and at angles of 0/180, 90, and 45 degrees. Each pole was equipped with two FIGHTLIGHT sensors (ten sensors in total), with the upper sensor being 85 cm from the floor and the lower 42 cm from the floor [8]. In a “ready/active” stance, participants were instructed to step and hit each sensor as it illuminated and return to the starting point as quickly as possible. Upon successful deactivation, participants were given 1 s to return to the starting point and reestablish their central gaze before the next light illuminated. Each light sensor illuminated two times for a total of 20 responses. Lights sensors were illuminated at random for every attempt. Participants completed three total attempts for each condition with 3-minute rest periods in between. Average response times from the single fastest performance were used for subsequent analysis.

### 2.4. Experiment 1

**Figure 2:**
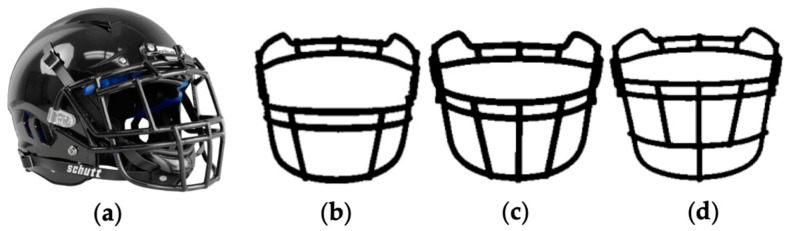
Headgear used in EXP1. (**a**) Schutt™ Custom Vengeance Pro Helmet, (**b**) Light-reinforced facemask (V-ROPO-TRAD), (**c**) Medium-reinforced facemask (V-ROPO-SW-TRAD), and (**d**) Heavy- reinforced facemask (VR-JOP-DW-TRAD).

**Figure 3:**
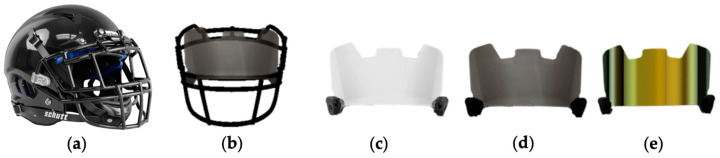
Headgear used in EXP 2. (**a**) Schutt™ Custom Vengeance Pro Helmet, (**b**) Light-reinforced facemask (V-ROPO-TRAD) depicted with visor, (**c**) Elitetek clear football visor (90%+ visual light transmittance; VLT), (**d**) Elitetek smoke-tinted football visor (48% VLT), and (**e**) Elitetek mirror-tinted football visor (28% VLT). Note: Lower VLT values indicate less passage of light through the visor.

**Figure 4:**
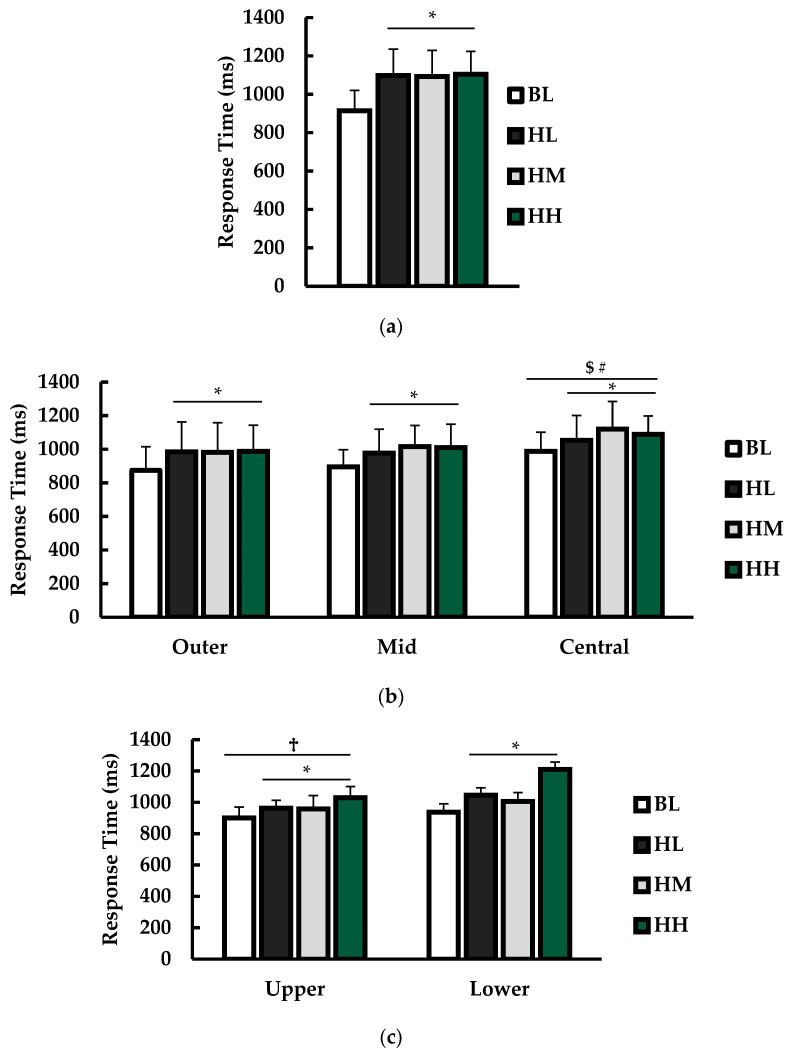
Response times between baseline (BL; white), helmet + light (HL; black), helmet + medium (HM; gray), and helmet + heavy (HH; green) conditions. (**a**) Overall response times (ms) over the drill. (**b**) Response times (ms) for each condition according to the sensor position. (**c**) Response times (ms) for each condition according to the sensor level. * indicates significantly different than BL (*p* < 0.05). # indicates significantly different from Mid (*p* < 0.05). $ indicates significantly different than outer (*p* < 0.05). † indicates significantly different than lower (*p* < 0.05).

EXP1 was completed in a single visit to an indoor fitness laboratory. The headgear used in EXP1 is shown in Figure 2. The same helmet shell was used for all experimental testing (Vengeance Pro LTD, Schutt; Litchfield, IL, USA; Figure 2a). In EXP1, participants completed the previously described dynamic visuomotor tests under the following conditions: (1) baseline (BL; no helmet), (2) helmet + light-reinforced facemask (HL; Figure 2b), (3) helmet + medium-reinforced facemask (HM; Figure 2c), and (4) helmet + heavy-reinforced facemask (HH; Figure 2d). Conditions were randomized to control for possible learning effects or testing fatigue [6,9]. Facemasks were exchanged during rest periods between conditions. The categorization of facemask reinforcement was adapted from previous work using similar headgear equipment [6,7]. 

### 2.5. Experiment 2

EXP2 was completed on a second visit separate from EXP1 in the same indoor fitness laboratory. The headgear used in EXP2 is shown in Figure 3. The same helmet shell (Figure 3a) and light-reinforced facemask (Figure 3b) were used for all experimental testing (Vengeance Pro LTD, Schutt; Litchfield, IL, USA; Figure 3a). In EXP2, participants completed the previously described dynamic visuomotor tests under the following conditions: (1) baseline (BL; no helmet), (2) helmet + clear visor (HCV; Figure 3c), (3) helmet + shaded visor (HSV; Figure 3d), (4) helmet + mirrored visor (HMV; Figure 3e). Conditions were randomized to control for possible learning effects or testing fatigue [6,9]. Visors were equipped with quick-release clips (Underamour, Baltimore, MD, USA) and exchanged during rest periods between conditions. Visors (Elitetek, Waterloo, IL, USA) were identical except for differences in visual light transmittance (VLT). The clear visor provided approximately 90% + VLT, the shaded visor provided 48% VLT, and the mirrored visor provided 28% VLT [6]. The categorization of visors was adapted from previous work using similar headgear equipment [6,7]. 

### 2.6. Data Analysis

Data analysis was completed utilizing Jamovi software (Version 0.9; Jamovie, Sydney, Australia). For both experiments, response times were analyzed using the overall response, position of the sensor (i.e., central, mid, outer), and level of the sensor (i.e., upper, lower). Due to the same design and number of conditions, identical statistical approaches were used for EXP1 and EXP2. 

To analyze overall response times, a 1 × 4 [group × condition] repeated-measures ANOVA was utilized. For the angle of view, the central and average of both mid/outer pole times were used for analysis. A 3 × 4 [position × condition] repeated-measures ANOVA was conducted for analysis. For the level of view, average response times from all sensors in the upper and lower levels were used for analysis. A 2 × 4 [level × condition] repeated-measures ANOVA was used for analysis. To detect differences in means and correct for multiple comparisons, a Bonferroni–Holm post-hoc test was used when significant main effects were detected. Estimates of effect size for main effects were calculated using eta squared (η^2^) and interpreted as follows: 0.01—small; 0.06—medium; and ≥0.14—large [15,16]. Significance was set at *p* ≤ 0.05. All data are presented as mean ± standard deviation (SD).

## 3. Results

### 3.1. Experiment 1 (Facemasks)

Response time results from EXP1 are presented in Figure 4. 

#### 3.1.1. Overall

For overall average response time (ms; Figure 4a), there was a main effect for condition (*p* < 0.001; η^2^ = 0.091). Multiple comparisons revealed that BL response time was significantly faster than HL (*p* = 0.001), HM (*p* < 0.001), and HH (*p* < 0.001) conditions. No other significant differences were observed, although there was a trend toward significance for HM response times being faster than HH (*p* = 0.062).

#### 3.1.2. Sensor Position (Outer, Mid, and Central)

For response times (ms) from different sensor positions (Figure 4b), there was a main effect for condition (*p* < 0.001; η^2^ = 0.091) and position (*p* < 0.001; η^2^ = 0.087). There was no interaction between condition and position (*p* = 0.548; η^2^ = 0.004). Multiple comparisons revealed that BL response time was significantly faster than HL (*p* = 0.001), HM (*p* < 0.001), and HH (*p* < 0.001) conditions. For position, response time was significantly faster in outer (*p* < 0.001) and mid (*p* < 0.001) positions compared to central. There was no difference between the outer and mid positions (*p* = 0.310). 

#### 3.1.3. Sensor Level (Upper and Lower)

For response times (ms) from different sensor levels (Figure 4c), there was a main effect for condition (*p* < 0.001; η^2^ = 0.133) and sensor level (*p* = 0.041; η^2^ = 0.092). There was no interaction between condition and level (*p* = 0.420; η^2^ = 0.008). Compared to BL, response times were significantly slower for HL (*p* = 0.001), HM (*p* < 0.001), and HH (*p* < 0.001) conditions. Response times for the upper sensors were significantly faster than the lower sensors (*p* = 0.041).

### 3.2. Experiment 2 (Visors)

**Figure 5:**
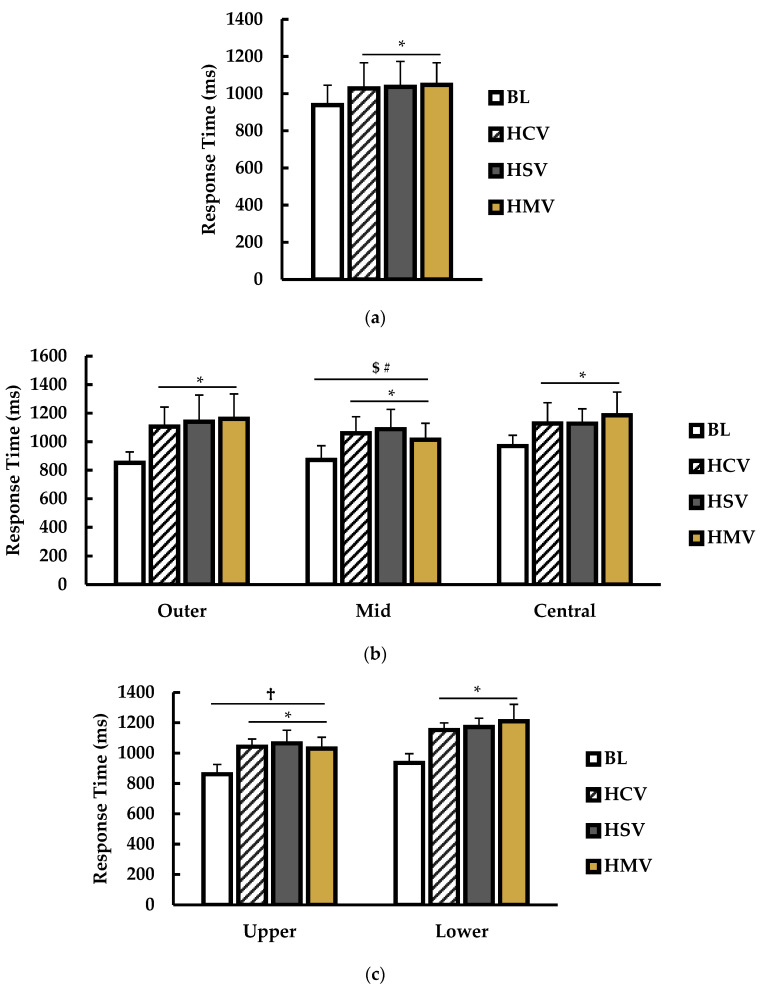
Response times between baseline (BL; white), helmet + clear visor (HCV; stripe), helmet + smoke visor (HSV; gray), and helmet + mirrored visor (HMV; gold) conditions. (**a**) Overall response times (ms) over the drill. (**b**) Response times (ms) for each condition according to the sensor position. (**c**) Response times (ms) for each condition according to the sensor level. * indicates significantly different than BL (*p* < 0.05). # indicates significantly different from central (*p* < 0.05). $ indicates significantly different than outer (*p* < 0.05). † indicates significantly different than lower (*p* < 0.05).

Response time results from EXP2 are presented in Figure 5. 

#### 3.2.1. Overall

For overall average response time (ms; Figure 5a), there was a main effect for condition (*p* < 0.001; η^2^ = 0.321). Multiple comparisons showed that BL response time was significantly faster than HCV (*p* = 0.001), HSV (*p* < 0.001), and HMV (*p* < 0.001) conditions. No other significant differences were noted.

#### 3.2.2. Sensor Position (Outer, Mid, and Central)

Response times (ms) from different sensor positions (Figure 5b) showed a main effect for condition (*p* < 0.001; η^2^ = 0.231) and position (*p* < 0.001; η^2^ = 0.054). There was a significant interaction between condition and position (*p* < 0.001; η^2^ = 0.037). Multiple comparisons revealed that BL response time was significantly faster than HCV (*p* < 0.001), HSV (*p* < 0.001), and HMV (*p* < 0.001) conditions. For position, response time was significantly faster in mid compared to outer (*p* < 0.001) and central (*p* < 0.001) positions. There was no difference between outer and central positions (*p* = 0.350). 

#### 3.2.3. Sensor Level (Upper and Lower) 

For response times (ms) from different sensor levels (Figure 4c), there was a main effect for condition (*p* < 0.001; η^2^ = 0.461) and sensor level (*p* < 0.001; η^2^ = 0.189). There was no interaction between condition and level (*p* = 0.071; η^2^ = 0.013). Compared to BL, response times were significantly slower for HCV (*p* < 0.001), HSV (*p* < 0.001), and HMV, (*p* < 0.001) conditions. Response times for the upper sensors were significantly faster than the lower sensors (*p* < 0.001).

## 4. Discussion

Existing research has established that AF headgear alters athletes’ visual field and reaction time performance. Recently, heavier reinforced facemasks and visors with low VLT have been linked to impaired visuomotor performance, particularly in peripheral fields of view. However, nearly all these studies to date have utilized laboratory visuomotor tests where the athlete is stationary, which is less translatable to movements reliant on visuomotor ability during practice and gameplay. The current study sought to build upon this evidence by testing various degrees of facemask reinforcement and visor VLT using a visuomotor drill reliant on gross movement that may be more realistic to gameplay compared to being stationary. To this end, EXP1 showed that regardless of facemask reinforcement, response times were worsened under helmeted conditions compared to BL. This was apparent in all fields of view, although the response time was slower when responding to lower-positioned sensors compared to upper-positioned sensors. EXP2 showed that regardless of visor VLT, response times were worsened under visor conditions compared to the BL. Similarly, this was apparent in all fields of view, with lower-positioned sensors being worse regardless of the condition compared to upper-positioned sensors. These findings suggest that AF headgear equipped with facemasks and/or visors impair visuomotor performance in NCAA Division I players. 

### 4.1. Experiment 1 (Facemasks)

In EXP1, all helmeted conditions, regardless of facemask reinforcement, impaired response times compared to baseline without headgear. This is in support of previous findings showing impaired reaction time with AF headgear. Miller et al. showed impaired simple reaction time while wearing protective AF headgear, deficits of which favored peripheral fields [9]. Similarly, Creamer et al. and Ballmann et al. showed impairments in simple reaction time with AF headgear but specifically found that heavier reinforcement of facemasks resulted in exacerbation of impairment [6,7]. Current findings support the notion that AF headgear impairs reactive ability, but disparities between findings lie in the lack of present changes in response time from varying facemask reinforcement. While the reasons for disparities are not fully apparent, they may be due to differences in the stationary versus dynamic nature of the tests. In previous studies examining facemask reinforcement, players were instructed to maintain the same gaze and head position throughout. However, during the current dynamic drill, players were able to make head adjustments since they were performing more gross movements. It is plausible the disparities between the extremes of impairment are due to compensation from neck and head movement. Previous evidence has shown that heavier facemasks may lead to greater head impact, specifically on the top of the head [17]. This would suggest that heavier gauged facemasks cause players to change their neck and head angle upon impact, which may be related to visibility from the heavier gauge facemask. Thus, the current lack of differences in response time between facemask reinforcement may be due to head or neck movement that is required to compensate for the lack of viability. Indeed, previous work has shown that AF headgear causes postural changes to the neck and head, albeit it is unknown if lack of visibility is the sole source of head and neck movement modifications. Such decrements are not unique to AF as other sports have noted similar findings of vision impairment with protective headgear [10,18]. From a practical standpoint, this has immense implications for safety and head injuries. It is widely believed that concussions and head injuries are due to poor tackling form and/or poor coaching of proper technique. While largely speculative at this time, current findings, along with others showing vision impairment when the neck and head are stationary, may implicate AF headgear as a contributor to head and neck injuries due to visual impairment. From a performance standpoint, visuomotor performance and motion perception have been connected to AF-specific skills, including catching and interceptions [19]. Thus, the reduced visuomotor ability with AF headgear may translate to worsened reactive ability to the ball and/or to other players on the field during gameplay. However, the reader is cautioned that these are mere points of discussion as of now, and data from the current investigation cannot give standalone support to the notion of head or neck movement as a compensatory action because of impaired visual field. Additional investigations using eye-tracking and precise head tilt and position changes during movement are warranted. 

Interestingly, the position of sensors that appeared to be most negatively affected lay within the central versus outer or mid positions. This is in direct opposition to previous evidence showing that outer or peripheral visual fields are most impacted by heavier reinforced facemasks. For example, Creamer et al., Miller et al., and Ballmann et al. all showed that the primary views that are impaired by AF headgear are peripheral fields [6,7,9]. At this time, there is not a clear mechanism suggesting that wearing AF protective headgear results in impaired visuomotor in central views compared to peripheral. Previous studies have suggested that reacting to visual stimuli in peripheral fields becomes faster as central tasks become increasingly complex and cognitively demanding task [20]. Regarding the current findings, players may have devoted greater cognitive resources to reacting to peripheral sensors, thereby detracting attention and focus from central views. This is supported by theories suggesting that individuals will prioritize complex tasks over simple ones. Thus, it is feasible that more attentional focus and cognitive resources are required to react to peripheral fields, which could detract from response times to stimuli from the central view despite the purported lower cognitive demand. Further testing with more sophisticated movement-tracking techniques and diverse populations will be needed to support this further. 

### 4.2. Experiment 2 (Visors)

In EXP2, all helmet + visor conditions, regardless of VLT, impaired response times compared to baseline without headgear. This partially supports previous findings showing that shaded and mirrored visors impair reaction time performance in collegiate football players [6]. However, multiple investigations have shown that clear or near 100% VLT visors did not impair peripheral reaction time, while the current findings found opposing evidence [6,9]. Given the similar response between all visors, it is more likely than not that the presence of helmet + facemask in the experiment is what may have been the root cause of the impairment. Supporting this further, similar response times were seen between visor conditions for sensor position and level. Thus, it may be tempting to conclude from present findings that the addition of visors, namely shaded or mirrored ones, does not add decrement to visuomotor ability above that of a helmet + facemask. While current findings support this conclusion, it is worth noting that shaded and tinted visors are primarily employed, when approved by regulatory agencies, for light sensitivity or other ocular conditions. Indeed, optometric guidelines have been implemented to establish a quantifiable clinical need for tinted or mirrored visors [11]. The current experiment was conducted indoors, which controls for ambient lighting across conditions to allow for proper comparison. However, the real-world rationale for the implementation of these visors often involves blocking bright light from the sun or overhead flood lights. It is plausible that the lack of differences between visor conditions was due to the standardization of light at standard indoor lumen intensities. Since visors with lower VLT are primarily used to block extraneous light stimuli, apparent benefits and/or decrements may not have been evident. Proper field tests utilizing visors in natural outdoor or overhead flood lighting are sorely needed although the feasibility of completing such a study is admittedly complicated by variations in the external environment. However, EXP2 conducted in the current controlled environmental setting suggests that the presence of the helmet + facemask itself appears to be causing the greatest impairment. This is bolstered by findings from EXP1 showing impairment with the same helmet + facemask and previous studies also utilizing the same equipment [6,7]. It may be possible that the addition of a visor does not result in any additional need for compensatory head or neck movement. Thus, no benefits or impairments were noted with any visor type aside from the documented decrement while wearing solely the helmet + facemask. 

### 4.3. Limitations and Conclusions

Despite the novel findings and testing battery utilized currently, there were some limitations to the current study. First, while the current visuomotor drill is more similar to gameplay than previous investigations using stationary testing, it was still performed in a controlled laboratory setting. Factors such as ambient temperature, humidity, helmet make/model, playing experience, etc., that are commonly experienced or accounted for on the field cannot be ruled out as influencing findings. Furthermore, head and/or neck movement was not quantified or controlled during the drill. This was in part because head and neck movement are key fundamentals for players in gameplay, and limiting or controlling for this may have decreased application potential and thus was not. However, future studies using inertial movement units (IMUs) should investigate real-time head and neck tilt to identify the degree of compensatory movement resulting from poor vision. Lastly, participants completed the drill in a non-fatigued state which is unlike that of typical AF gameplay. Since response times and visuomotor performance tend to decrease with fatigue onset [21], it is still unknown what severity present impairments will have on performance and safety on the field. In conclusion, AF headgear impairs visuomotor drill performance regardless of facemask reinforcement or visor VLT. While these support previous findings, impairment in response times was comparatively less noticeable between conditions, which may indicate a role for compensatory head and neck movement to ensure proper lines of sight. Future field research accounting for differences in head and neck movement is needed.

## Data Availability

All data are contained within the manuscript.
